# Peripapillary Choroidal Neovascularization Type 2 With Pitchfork Sign: A Case Report

**DOI:** 10.18502/jovr.v18i4.14557

**Published:** 2023-11-30

**Authors:** Yousef Alizadeh, Maryam Dourandeesh

**Affiliations:** ^1^Eye Research Center, Department of Eye, Amiralmomenin Hospital, School of Medicine, Guilan University of Medical Sciences, Rasht, Iran; ^4^Yousef Alizadeh: https://orcid.org/0000-0001-5196-4166

**Keywords:** Choroidal Neovascularization Type 2, Optical Coherence Tomography, Peripapillary Choroidal Neovascularization, Pitchfork Sign

## Abstract

**Purpose:**

This study aimed to report a case of peripapillary choroidal neovascularization (CNV) with a pitchfork sign.

**Case Report:**

A young female presented with a progressive and painless visual blurring of the left eye. Ophthalmoscopic findings and results of optical coherence tomography (OCT), OCT angiography (OCTA), and fluorescein angiography (FAG) were evaluated. OCT showed subretinal hyperreflective material adjacent to the optic nerve head with multiple vertical finger-like projections extending into the outer retina (pitchfork sign). OCTA revealed that seafan-shaped high-flow vessels above the retinal pigment epithelium (RPE) were compatible with CNV type 2 with a large feeder vessel completely contiguous with the optic nerve. No evidence of ocular or systemic inflammation was found.

**Conclusion:**

Pitchfork sign can be seen in CNV type 2 in either inflammatory or noninflammatory conditions.

**Figure 1:**
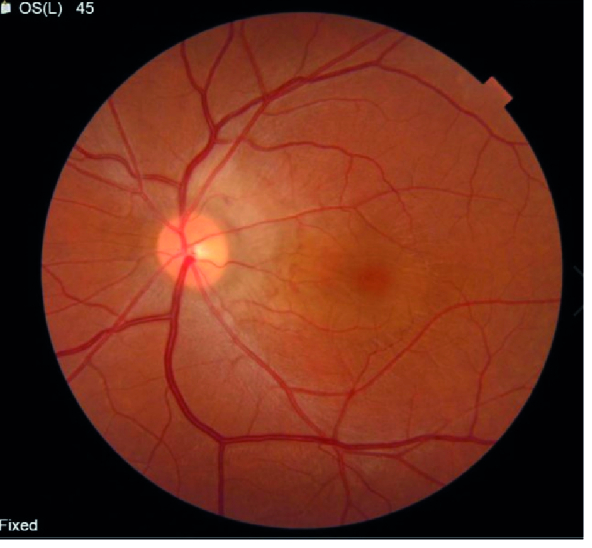
Color fundus photograph (Left eye).

**Figure 2:**
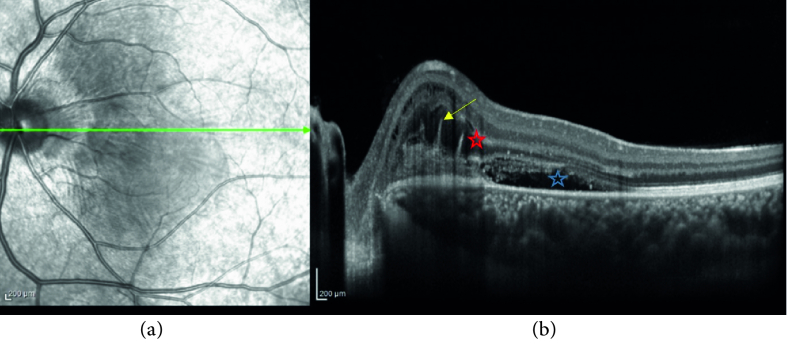
(a) Infrared fundus photograph (left eye) (b) optical coherence tomography (OCT) of the left eye. Hyperreflective finger-like projections (pitchfork sign) (yellow arrow), disruption of ellipsoid zone (EZ) over the lesion (red asterisk), and remarkable subretinal fluid (blue asterisk).

**Figure 3:**
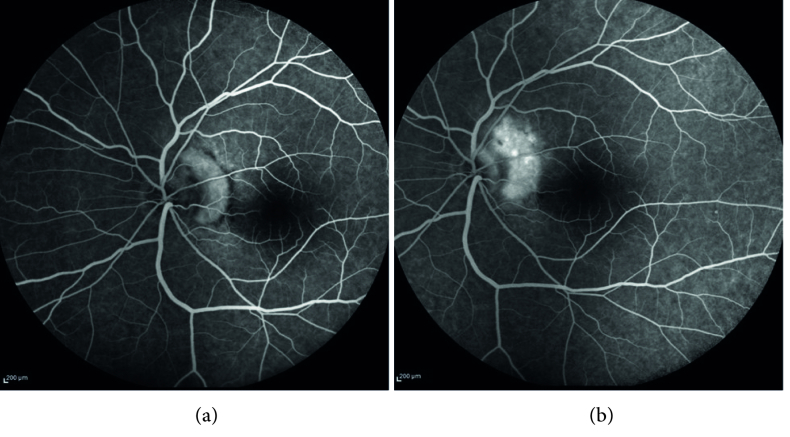
Fluorescein angiography (left eye, early and late phase): (a) early well-defined hyperfluorescence (b) late leakage.

**Figure 4:**
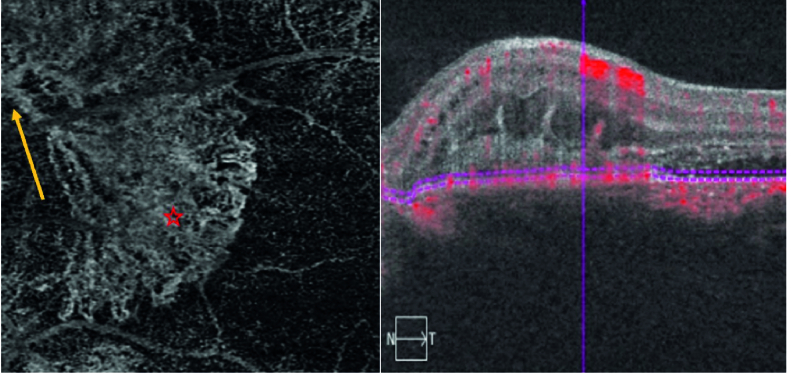
OCTA (outer retina segmentation): “seafan” shaped high flow vessels above the RPE entwined with a dense network of finer vessels (red asterisk) compatible with CNV type 2, a large feeder vessel (yellow arrow), no vascular flow within the vertical projections except in one at the temporal border of the lesion.

##  INTRODUCTION

Choroidal neovascularization (CNV) is classified into three types according to the optical coherence tomography (OCT) findings. In CNV type 1, pathological vessels grow under retinal pigment epithelium (RPE), whereas in CNV type 2 these vessels penetrate the RPE and proliferate into the subretinal space. CNV type 3 originates in the deep retinal capillary plexus (DCP); therefore, it is an intraretinal lesion.^[1,2,3]^ It should be noted that CNV type 2 can be observed in several ocular diseases, such as age-related macular degeneration, myopic macular degeneration, and punctate inflammatory choroidopathy (PIC)/multifocal choroiditis (MFC).^[3,4,5]^ The pitchfork sign was first introduced by Hoang et al as a distinct finding of CNV type 2 in inflammatory conditions and was described as multiple, distinctive, vertical finger-like projections extending from the area of active CNV into the outer retina.^[3]^ The inflammatory conditions associated with this sign include PIC/MFC, choroidal osteoma, and intraocular tuberculosis.^[3,7,8,9]^ Later on, the pitchfork sign was described to appear in eyes without any sign of ocular inflammation.^[6]^ In all these reports, the CNV lesion has been farther away from the optic disc. Herein, we report a case of peripapillary CNV type 2 with a pitchfork sign that had no sign of ocular or systemic inflammation.

##  CASE PRESENTATION

A 33-year-old female presented to our hospital with a progressive and painless visual blurring of the left eye for one month prior to a visit by the ophthalmologist. The patient had no history of ocular surgery or trauma. Moreover, the patient's past ocular, past medical, and drug use history were negative. The best-corrected visual acuity (BCVA) was 20/20 and 20/25 in the right and left eyes, respectively. The results of slit lamp examination were unremarkable and no vitreous cells were observed. Intraocular pressure (IOP) was 17 mm Hg (OU). Fundus examination of the left eye revealed peripapillary grayish subretinal thickening as well as subretinal fluid temporal to the lesion in the center of the macula. Peripheral retinal examination demonstrated no lesion [Figure 1]. The fundus examination of the right eye was normal.

The macular optical coherence tomography (OCT) of the left eye showed hyperreflective material located in the subretinal space above the RPE adjacent to the optic nerve head with multiple vertical finger-like projections extending into the outer retina (pitchfork sign). There was a disruption of the ellipsoid zone (EZ) over the lesion and remarkable subretinal fluid extending from the lesion into the fovea was present [Figure 2]. Fluorescein angiography (FAG) findings included early well-defined hyperfluorescent lesions with late leakage [Figure 3]. Optical coherence tomography angiography (OCTA) revealed seafan-shaped high-flow vessels above the RPE compatible with CNV type 2. A large feeder vessel completely contiguous with the optic nerve was observed. It is worth mentioning that there was no vascular flow within the vertical projections except in one at the temporal border of the lesion [Figure 4].

The complete blood count (CBC), sedimentation rate, C-reactive protein, syphilis serology, purified protein derivative (PPD), chest X-ray, autoimmunity markers (ANA, RF, and ACE), as well as rheumatology consult, suggested no sign of abnormality.

##  DISCUSSION 

There are a few reports of characteristic pitchfork signs in patients with CNV type 2.^[3,6,7,8,9]^ In all these reports the lesion is farther away from the optic nerve head and in the majority of them, there is an inflammatory background. In the present case study, we introduced a case of peripapillary CNV type 2 with a pitchfork sign without any underlying etiology.

In our patient, we found the pitchfork sign through OCT; however, we did not find any sign of ocular or systemic inflammation or any other underlying etiology. In OCT, the pitchfork sign was first described by Hoang et al^[3]^ to distinguish inflammatory CNV from other causes of neovascularization type 2. They hypothesized that when sub-RPE hyperreflective non-neovascular PIC/MFC lesions progress into neovascular lesions, the material over the RPE may show the characteristic pitchfork sign. They also hypothesized that this finding may be due to the presence of other inflammatory materials, such as fibrin.^[3]^ Rajabian et al and Berensztejn et al reported pitchfork signs in cases with choroidal osteoma. They proposed that inflammation is the most important stimulus for the development of CNV in these patients.^[7,8]^ Ramtohul et al showed pitchfork signs in a known case of intraocular tuberculosis.^[9]^ In line with previous reports, the cause was underlying inflammation. On the other hand, Falavarjani et al described this sign in five eyes without any sign of intraocular inflammation, and there were no underlying etiology in two out of these five eyes. They speculated that traction of type 2 CNV complex on the outer retinal layers and consequent dragging of the layers or Muller cell activation could explain the presence of the pitchfork sign.^[6]^ Subsequently, this pattern resolved following the treatment that resulted in the regression of CNV type 2 to CNV type 1.

In our case, the CNV lesion was completely adjacent to the optic nerve head and the feeder vessel was completely contiguous with it. In all previous reports, CNV type 2 was either adjacent to or beneath the fovea and farther away from the optic disc.^[3,6,7,8,9]^ The CNV type 2 can involve the macula, peripapillary, or other parts of the fundus. Traction of the CNV on retinal layers or Muller cell activation justifies the presence of the pitchfork sign in CNV type 2 in any region. The rarity of this sign might be the reason why it has not been previously reported in any studies on the peripapillary area. In summary, the pitchfork sign can be seen in association with peripapillary CNV type 2 in either inflammatory or noninflammatory conditions and in any region where CNV type 2 may be present.

##  Financial Support and Sponsorship

None.

##  Conflicts of Interest

None.
